# BASP1 expression is associated with poor prognosis and is correlated with immune infiltration in gastric cancer

**DOI:** 10.1002/2211-5463.13654

**Published:** 2023-06-04

**Authors:** Tao Wang, Xiaojing Liu, Tong Wang, Lei Zhan, Mingjun Zhang

**Affiliations:** ^1^ Department of Oncology The Second Affiliated Hospital of Anhui Medical University Hefei China; ^2^ Department of Obstetrics and Gynecology The Second Affiliated Hospital of Anhui Medical University Hefei China; ^3^ Department of General Medicine The Second Affiliated Hospital of Anhui Medical University Hefei China

**Keywords:** BASP1, gastric cancer, immune microenvironment, prognostic marker

## Abstract

BASP1 is a membrane‐bound protein that plays a promotional or inhibitory role in a variety of tumors; however, its role in gastric cancer (GC) and in the immune microenvironment has not been reported. The objectives of this study were to determine whether BASP1 is a valuable prognostic marker for GC and to explore its role in the immune microenvironment of GC. The expression level of BASP1 in GC was analyzed based on the TCGA dataset and further verified using GSE54129 and GSE161533 datasets, immunohistochemistry, and western blotting. The association between BASP1 and clinicopathological characteristics, as well as its predictive value, were examined using the STAD dataset. Cox regression analysis was performed to determine whether BASP1 can be used as an independent prognostic indicator for GC, and a nomogram was constructed to predict OS. The association between BASP1 and immune cell infiltration, immune checkpoints, and immune cell markers was confirmed by enrichment analysis, as well as analysis based on the TIMER and GEPIA databases. BASP1 was observed to be highly expressed in GC and was associated with a poor prognosis. The expression of BASP1 was positively correlated with the expression of immune checkpoints and immune cell markers, as well as immune cell infiltration. Thus, BASP1 may serve as a standalone prognostic indicator for GC. BASP1 is highly correlated with immune processes, and its expression is positively correlated with the degree of immune cell infiltration, immune checkpoints, and immune cell markers.

## Graphical abstract

BASP1 is highly expressed in gastric cancer and can be used as an independent indicator to predict poor overall survival in patients with gastric cancer. BASP1 is highly correlated with immune processes, and its expression is positively correlated with the degree of immune cell infiltration, immune checkpoints, and immune cell markers.

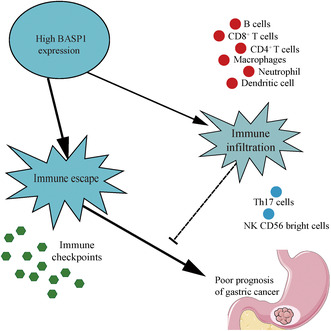

**Abbreviations**
ACC: adenoid cystic carcinoma
BASP1: brain acid‐soluble protein 1
BLCA: bladder urothelial carcinoma
BRCA: breast invasive carcinoma
CESC: cervical squamous cell carcinoma and endocervical adenocarcinoma
CHOL: cholangiocarcinoma
COAD: colon adenocarcinoma
DEGs: differentially expressed genes
DLBC: lymphoid neoplasm diffuse large B‐cell lymphoma
DSS: disease‐specific survival
ESCA: esophageal carcinoma
GBM: glioblastoma multiforme
GC: gastric cancer
GEO: Gene Expression Omnibus database
GO: gene ontology
GSEA: gene set enrichment analysis
GTEx: genotype‐tissue expression
HNSC: head and neck squamous cell carcinoma
HPA: The Human Protein Atlas
KEGG: Kyoto Encyclopedia of Genes and Genomes
KICH: kidney chromophobe
KIRC: kidney renal clear cell carcinoma
KIRP: kidney renal papillary cell carcinoma
LAML: acute myeloid leukemia
LGG: brain lower grade glioma
LIHC: liver hepatocellular carcinoma
LUAD: lung adenocarcinoma
LUSC: lung squamous cell carcinoma
MESO: mesothelioma
OS: overall survival
OV: ovarian serous cystadenocarcinoma
PAAD: pancreatic adenocarcinoma
PCPG: pheochromocytoma and paraganglioma
PFI: progression‐free interval
PRAD: prostate adenocarcinoma
READ: rectum adenocarcinoma
ROC: receiver‐operating characteristic curve
SARC: sarcoma
SKCM: skin cutaneous melanoma
STAD: stomach adenocarcinoma
TCGA: The Cancer Genome Atlas
TGCT: testicular germ cell tumors
THCA: thyroid carcinoma
THYM: thymoma
UCEC: uterine corpus endometrial carcinoma
UCS: uterine carcinosarcoma
UVM: uveal melanoma

Globally, gastric cancer is the third most prevalent cause of cancer mortality and the fifth most common cancer [1]. Although the overall incidence and mortality of GC have been declining for half a century, the incidence of GC in younger age groups (under 50 years) is increasing [2]. The main risk factors for GC include *Helicobacter pylori* infection, high salt intake, low vegetable, and fruit fiber intake [1, 3], and in 10% of the cases, familial or genetic characteristics [1, 4]. Conventional treatments for GC include surgery, radiotherapy, and chemotherapy [5]. Although the use of targeted agents and immune checkpoint inhibitors has led to substantial improvements in the survival of patients with GC, single‐target therapy is not ideal [6] because of the high intra‐ and inter‐tumor heterogeneity of GC [7] and the complexity of the tumor microenvironment [8, 9]. Therefore, the development of novel treatment targets and prognostic indicators is crucial.

Brain acid‐soluble protein 1 (BASP1), also known as NAP22 or CAP‐23, was originally thought to be a membrane‐bound protein belonging to the family of neuronal growth‐associated proteins [10] and is particularly abundant in nerve endings. It was subsequently found in other types of tissues and cells. In addition to its involvement in promoting axonal growth, regeneration, and remodeling [11], BASP1 directly interacts with cholesterol to mediate chromatin remodeling [12, 13] and represses the function of transcription factors, including c‐myc and WT1 [14, 15]. Its function in apoptosis and differentiation has also been reported [16]. BASP1 has been found to be downregulated in lymphocytic leukemia [17, 18] and inhibits cell proliferation in acute myeloid leukemia and thyroid cancer cells [19, 20]. BASP1 has also been shown to be downregulated in hepatocellular carcinoma via promoter methylation [21]. In breast cancer, BASP1 enhances the efficacy of tamoxifen by synergizing with the estrogen receptor alpha, and a better prognosis for survival is associated with increased BASP1 expression [22]. In addition, high expression of BASP1 allows patients with pancreatic cancer to benefit more from postoperative adjuvant chemotherapy [23]. These results suggested that BASP1 has a tumor‐suppressive effect. Recent studies have also reported that BASP1 expression is upregulated in several cancers, including lung cancer [24], cervical cancer [25], head and neck squamous cell carcinoma [26], and tongue squamous cell carcinoma [27], and is associated with poor prognosis. However, the role of BASP1 in GC and the immune microenvironment has not yet been documented.

In this study, we found that BASP1 is highly expressed in GC, acts as an independent prognostic indicator in GC, and is associated with poor prognosis by univariate and multifactorial Cox regression analysis, as well as analysis of clinicopathological characteristics and prognosis. Immune cell infiltration analysis revealed a significant positive correlation between the expression of BASP1 and the infiltration levels of immune cells, such as CD8+ and CD4+, as well as the expression of immune checkpoints such as PD‐1 and PD‐L1, suggesting that BASP1 may play an important regulatory role in GC tumor immunity.

## Materials and methods

### Data sources and differential gene expression analysis

The Cancer Genome Atlas (TCGA, https://portal.gdc.cancer.gov/) dataset (containing 33 cancers, 10 363 tumor samples, and 730 paracancer samples) and the normal tissue transcriptome dataset from GTEx (https://xenabrowser.net/datapages/; containing 4683 normal samples) were processed by the Toil process [28] and used for the analysis of BASP1 differential expression in pancancer (including 414 GC samples, 36 paraneoplastic samples, and 174 normal gastric tissue samples). It was also validated using GEO datasets GSE54129 (111 GC samples, 21 paraneoplastic samples) and GSE161533 (56 GC samples, 28 paraneoplastic samples). The protein expression of BASP1 in GC cell lines was analyzed using the Human Protein Atlas (HPA) database (www.proteinatlas.org) [29].

### Collection of specimens

GC and parietal tissues from 10 patients were collected from the Department of General Surgery, The Second Affiliated Hospital of Anhui Medical University. All patients were diagnosed with GC by histopathology, and those with a history of other malignancies were excluded. The study was conducted in accordance with the Declaration of Helsinki and approved by the Ethics Review Committee of the Second Affiliated Hospital of Anhui Medical University (YX2022‐075(F1)). Written informed consent was obtained from all the patients.

### Western blot

Proteins extracted from GC and paracancerous tissues were separated by 10% SDS/PAGE, transferred to PVDF membranes, and treated with anti‐human BASP1 (1 : 500; DF13578, Affinity, Changzhou, Jiangsu Province, China) and β‐ACTIN (1 : 5000, AF7018, Affinity) at 4 °C overnight. The membrane was incubated with the corresponding peroxidase‐linked secondary antibody for 1 h at 25 °C. Finally, the signal was detected using the ECL system. Intensity was measured using imagej software (National Institutes of Health, Bethesda, MD, USA).

### Clinical relevance and prognostic analysis

The correlation between differential expression of BASP1 in GC and age, sex, pathological stage, histological grade, and TNM stage was analyzed using r software (version 4.1.0) and tested using the Wilcoxon rank‐sum test. The r package ‘survival’ was used to analyze the relationship between BASP1 expression and overall survival (OS), progression‐free interval (PFI), and disease‐specific survival (DSS) in patients with GC. Independent prognostic indicators were determined using one‐way and multiway Cox regression analyses. On this basis, a nomogram was created to integrate multiple indicators for predicting 1‐, 3‐, and 5‐year OS, and was validated with calibration curves. The effectiveness of the OS predictive capability was verified using a receiver operating characteristic (ROC) curve.

### Screening for hub genes

Differentially expressed genes (DEGs) associated with the BASP1 gene in GC were analyzed using the LinkedOmics (http://www.linkedomics.org) database [30]. DEGs with correlation coefficients (cor) > 0.5 were then subjected to protein interaction network construction in the STRING (https://cn.string‐db.org/) database [31]. Moreover, 10 hub genes with the most interacting adjacency nodes were selected using cytoscape software, and enrichment analysis of GO function and KEGG pathway analysis were conducted [32].

### Immune cell infiltration analysis

The relationship between BASP1 expression and immune cell infiltration and somatic cell copy number in GC was analyzed using the TIMER (https://cistrome.shinyapps.io/timer/) database [33] and validated using the ssGSEA method for immune cell infiltration. The Kaplan–Meier plotter database (https://kmplot.com/analysis/) was used to analyze the correlation between BASP1 expression and prognosis of patients with GC at different levels of immune cell infiltration [34]. Correlation analysis between BASP1 and immune checkpoints and immune cell markers was performed using the TIMER and GEPIA (http://gepia.cancer‐pku.cn/) databases [35].

### Immunohistochemistry

After roasting at 65 °C for 30 min, the slides containing tissue were dewaxed in xylene for 20 min. Subsequently, the slides were hydrated with ethanol solution and pure water, placed in citrate buffer, and autoclaved for 2 min. After removing endogenous peroxidase with 90% methanol and 3% hydrogen peroxide solution, the slides were blocked with 1% bovine serum albumin at 25 °C for 30 min, followed by incubation with anti‐human BASP1 (1 : 150; DF13578, Affinity) for 12 h at 4 °C and secondary antibody for 30 min at room temperature. Finally, the slides were developed with diaminobenzidine (DAB) kit (CWBIO, Taizhou, Jiangsu Province, China), counterstained with hematoxylin, and observed under a microscope.

### Statistical analysis

Statistical analysis, data processing, and visualization in bioinformatics were performed using r software (version 4.1.0). The r packages involved include: ‘survival’, ‘rms,’ ‘pROC’, ‘ggplot2’, ‘clusterProfiler’, ‘org.Hs.eg.db’, and ‘GSVA’. The results of the western blot experiments were statistically analyzed using graphpad prism 9.0.0 program, and quantitative results were expressed as mean ± standard error of the mean. Statistical significance was set at *P* < 0.05.

## Results

### BASP1 is highly expressed in GC tissues

**Fig. 1:**
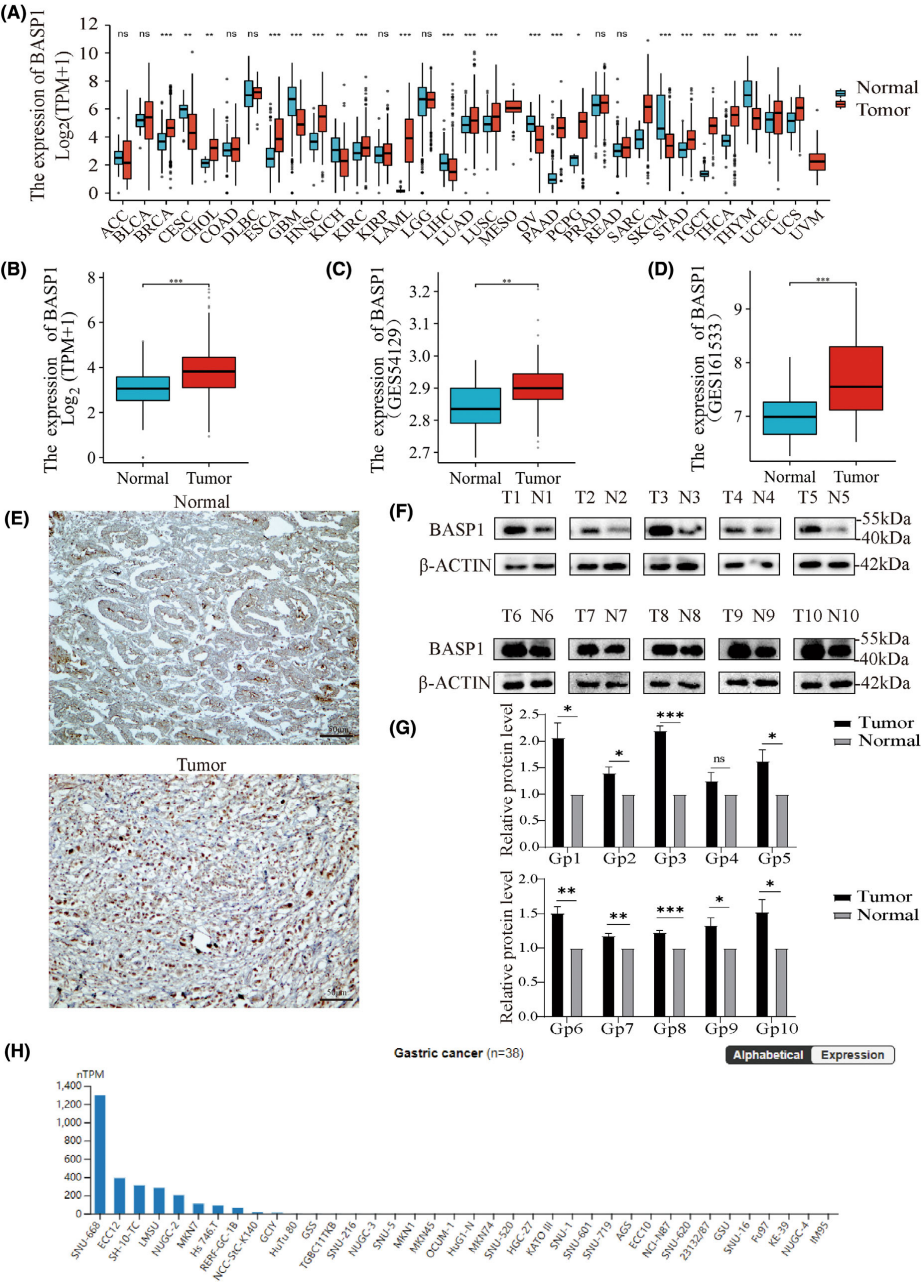
The expression of BASP1 is upregulated in GC. (A) Expression of BASP1 in different cancer types in TCGA combined with GTEx data. (B) Expression of BASP1 in gastric cancer in TCGA combined with GTEx data. (C) Expression of BASP1 in GSE54129 dataset. (D) Expression of BASP1 in GSE161533 dataset. (E) Immunohistochemical results of BASP1 expression in normal gastric and GC tissues (scar bar = 50 μm). (F, G) Expression of BASP1 protein in normal gastric tissues and GC tissues of clinical samples (mean ± SEM; independent sample *t*‐test; *n* = 10). (H) Expression of BASP1 mRNA in GC cell lines from HPA database. (*P < 0.05; **P < 0.01; ***P < 0.001; ns, no significant difference).

Pancancer data analysis based on TCGA and GTEx databases showed that, compared with that in normal tissues, the expression of BASP1 was significantly upregulated in 15 cancers, including GC (BRCA, CHOL, ESCA, HNSC, KIRC, LAML, LUAD, LUSC, PAAD, PCPG, STAD, TGCT, THCA, UCEC, and UCS). The expression was significantly downregulated in seven cancers (CESC, GBM, KICH, LIHC, OV, SKCM, and THYM; Fig. 1A,B). Subsequently, the GSE54129 and GSE161533 datasets verified that the expression of the BASP1 gene was markedly increased in GC (Fig. 1C,D). Immunohistochemical results also showed significantly higher protein expression of BASP1 in GC (Fig. 1E). In addition, western blot results of clinical samples confirmed the high expression of BASP1 in GC tissues (Fig. 1F,G). We then analyzed the mRNA expression of BASP1 in various GC cell lines using the HPA database, and the results are shown in Fig. 1H. The SNU‐668 had the highest expression while the IM95 had the lowest expression.

### Expression of BASP1 and clinicopathological characteristics of GC

**Fig. 2:**
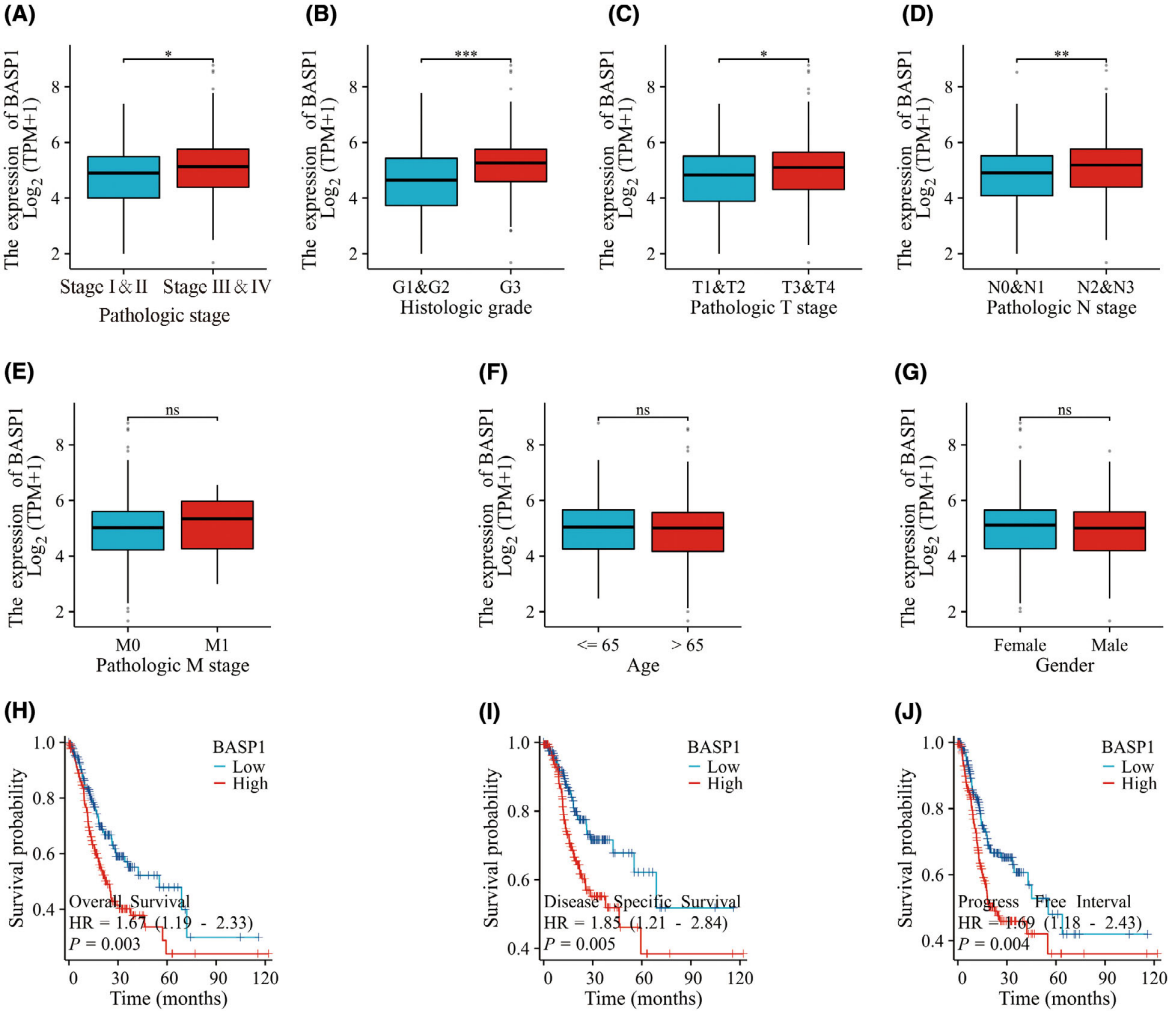
Relationship between the expression of BASP1 and clinicopathologic features of patients with GC. (A) The higher the pathological stage, the higher the expression of BASP1. (B) The higher the histological grade, the higher the expression of BASP1. (C) The higher the T stage the higher the expression of BASP1. (D) The higher the N stage, the higher the expression of BASP1. (E) The expression of BASP1 was independent of M staging. (F) The expression of BASP1 was independent of age (G) The expression of BASP1 was independent of gender. (H) The higher the expression of BASP1 the worse the OS of gastric cancer patients. (I) The higher the BASP1 expression the worse the DSS in gastric cancer patients. (J) The higher the expression of BASP1 the worse the PFI in gastric cancer patients. (The statistical method used in (A–G) is the Wilcoxon rank‐sum test, *n* = 367. The statistical method used in (H–J) is the Cox's proportional hazards regression model, *n* = 370. *P < 0.05; **P < 0.01; ***P < 0.001; ns, no significant difference).

Correlation analysis was used to determine the association between BASP1 expression and clinicopathological characteristics of patients with GC. The results showed that the expression of BASP1 was significantly different at different pathological stages, histological grades, T stage, and N stage (Fig. 2A–D), that is, the higher the pathological stage, histological grade, T stage, and N stage, the higher the expression of BASP1. However, differences in M stage, age, and sex were not significant (Fig. 2E–G).

### High expression of BASP1 is associated with poor prognosis in GC

**Fig. 3:**
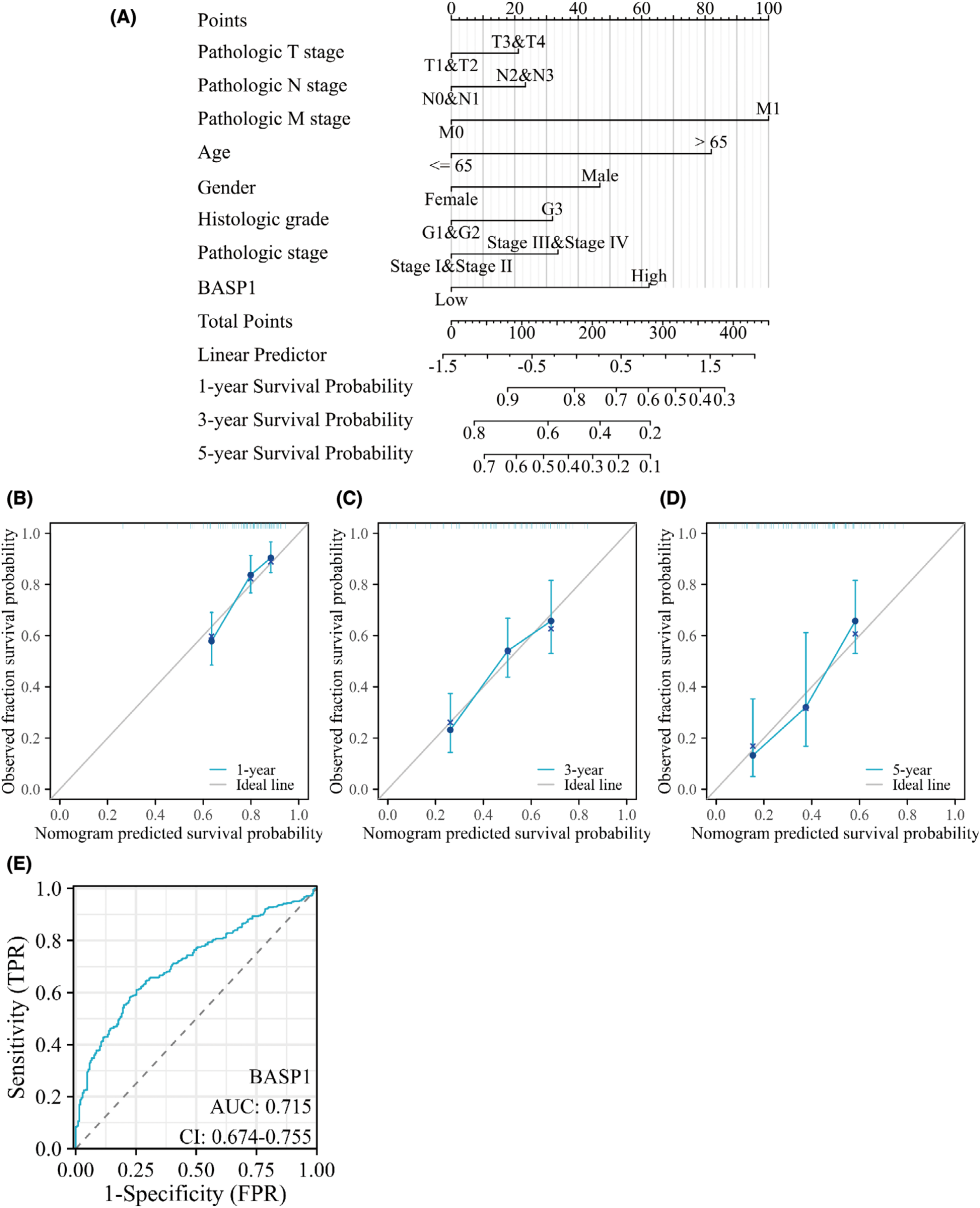
ROC curve and nomogram. (A) Nomogram to predict 1, 3, and 5 years of OS in patients with gastric cancer. (B–D) Calibration curves of 1‐, 3‐, and 5‐year OS forecast. (E) ROC curve.

Analysis based on the STAD dataset in TCGA database showed that patients with high BASP1 expression performed worse than those with low BASP1 expression in terms of OS, DSS, and PFI (Fig. 2H–J). In addition, univariate Cox regression analysis of clinicopathological variables in patients with GC showed that high expression of BASP1 was associated with poorer OS (Table 1). TNM stage, pathological stage, and age were also associated with poorer OS, while pathological grade was not statistically different from OS. Multifactorial Cox regression analysis showed that BASP1 could be an independent indicator in patients with GC, while M stage and age were equally distinguishable from other clinicopathological characteristics (Table 1). Subsequently, age, sex, TNM stage, pathological status, grade, and BASP1 expression were included in the scoring criteria to construct a nomogram for predicting the OS of patients with GC at 1, 3, and 5 years (Fig. 3A). The C‐index was 0.667 (95% CI: 0.641–0.694). The accuracy of the nomogram was confirmed by using a calibration curve. The results confirmed that the constructed nomogram was in good agreement with the actual observation and the prediction of OS in patients with GC (Fig. 3B–D), and it was almost free, portable, and intuitive in clinical application. Finally, the area under the ROC curve (AUC) was 0.715 (95% CI: 0.674–0.755), indicating that BASP1 expression was accurate in predicting the prognosis of patients with GC (Fig. 3E).

**Table 1:** Univariate/multivariate Cox regression analysis of BASP1 expression, OS, and clinicopathological characteristics in patients with GC.

Characteristics	Total (*N*)	Univariate analysis		Multivariate analysis	
		Hazard ratio (95% CI)	*P* value	Hazard ratio (95% CI)	*P* value
T stage	362				
T1&T2	96	Reference			
T3&T4	266	1.719 (1.131–2.612)	0.011	1.132 (0.673–1.905)	0.641
N stage	352				
N0&N1	204	Reference			
N2&N3	148	1.650 (1.182–2.302)	0.003	1.204 (0.751–1.932)	0.441
M stage	352				
M0	327	Reference			
M1	25	2.254 (1.295–3.924)	0.004	2.097 (1.063–4.137)	0.033
Pathologic stage	347				
Stage I&Stage II	160	Reference			
Stage III&Stage IV	187	1.947 (1.358–2.793)	<0.001	1.394 (0.795–2.445)	0.247
Age	367				
≤65	163	Reference			
>65	204	1.620 (1.154–2.276)	0.005	1.892 (1.293–2.768)	0.001
Histologic grade	361				
G1&G2	144	Reference			
G3	217	1.353 (0.957–1.914)	0.087	1.252 (0.841–1.862)	0.268
BASP1	370				
Low	186	Reference			
High	184	1.677 (1.201–2.341)	0.002	1.659 (1.148–2.397)	0.007

### BASP1 expression is closely associated with the immune system

**Fig. 4:**
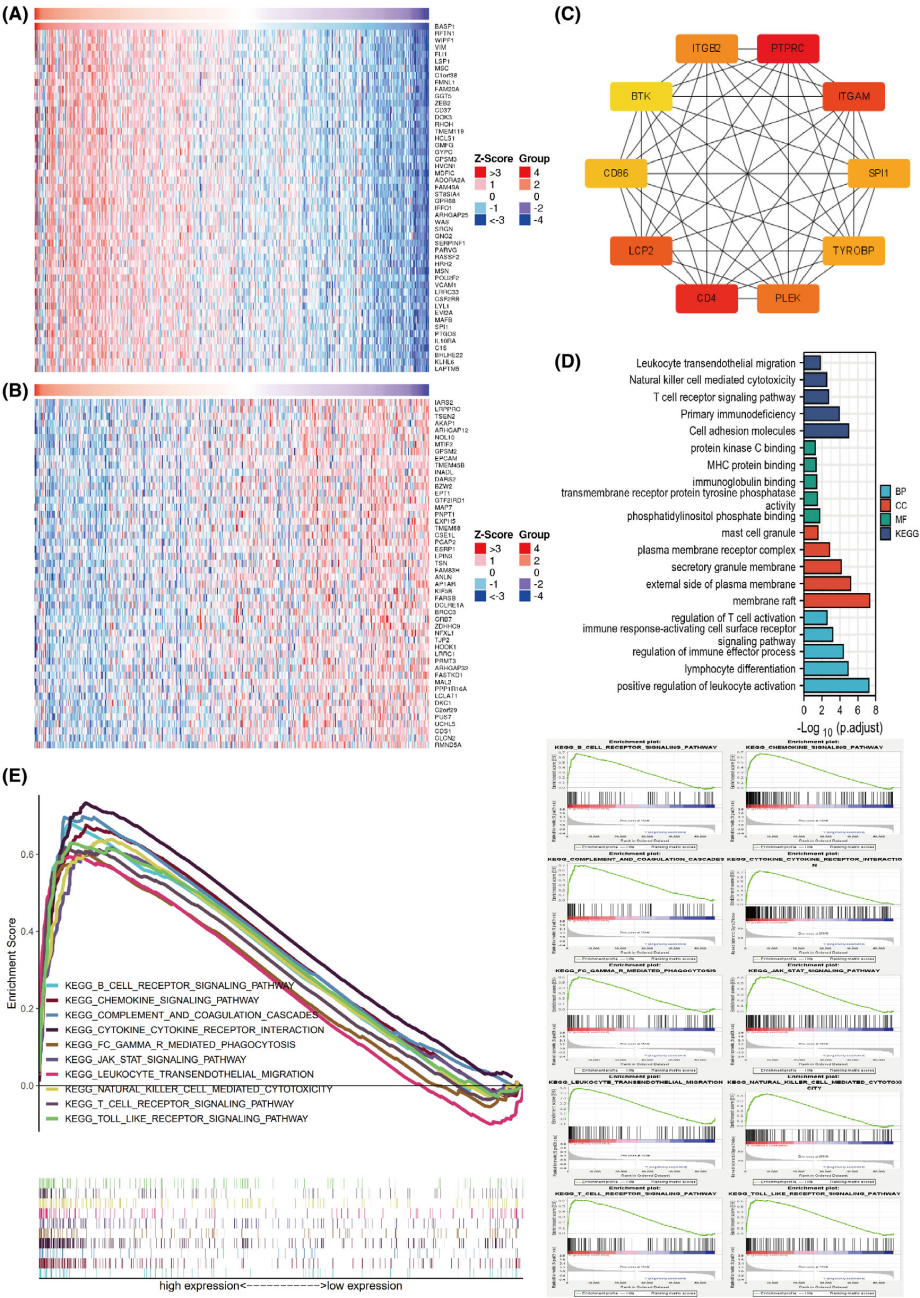
DEGs enrichment analysis and GESA enrichment analysis. (A) Heat map of differential genes positively associated with BASP1. (B) Heat map of differential genes negatively associated with BASP1. (C) HUB genes positively associated with BASP1. (D) GO and KEGG enrichment analysis of HUB genes. (E) GSEA enrichment analysis of the BASP1 high expression group showing the associated pathways.

A total of 587 DEGs associated with BASP1 (cor > 0.5) were identified by screening GC samples from TCGA dataset using the LinkedOmics website, and the top 50 DEGs in terms of correlation were visualized (Fig. 4A,B). Then, the interaction network among 587 proteins encoded by DEGs was constructed in the STRING database, and the 10 hub genes with the closest interaction relationship were identified (Fig. 4C). They were ITGB2, PTPRC, ITGAM, SPI1, TYROBP, PLEK, CD4, LCP2, CD86, and BTK. Subsequently, the GO functions and KEGG pathways enriched by hub genes were screened separately by enrichment analysis, and the results suggested that hub genes were mainly associated with the immune response (Fig. 4D). We also analyzed pathway enrichment in the high and low BASP1 expression groups using gsea software, and the results were similar to those of hub gene enrichment, showing that the pathways were mainly associated with the immune response (Fig. 4E).

**Fig. 5:**
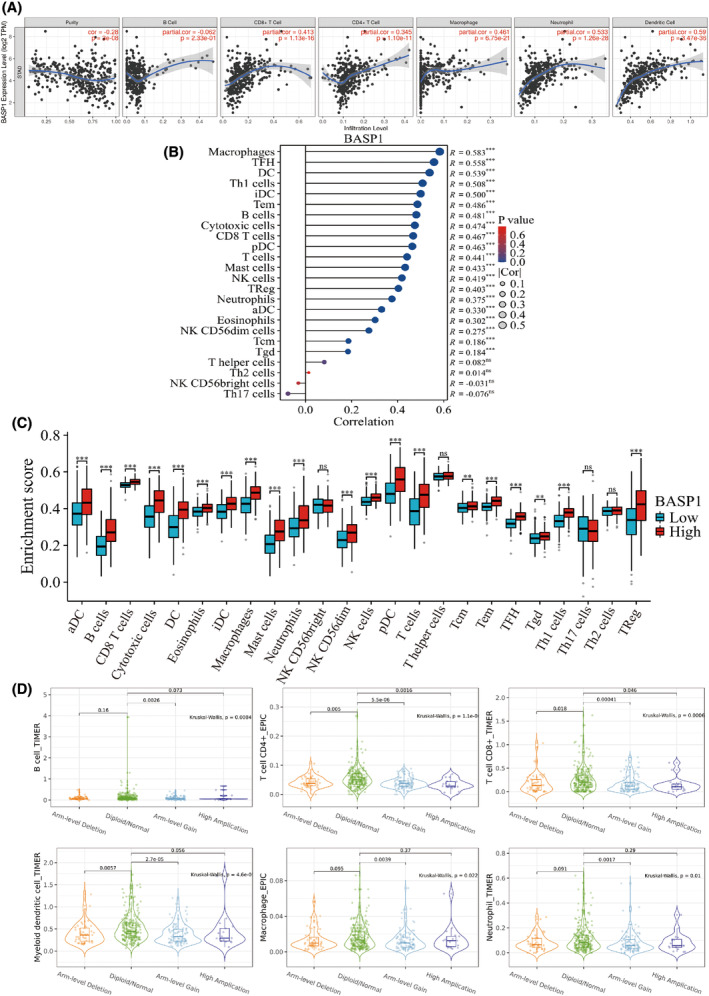
Correlation analysis between BASP1 expression and immune cell infiltration in GC. (A) Relationship between six immune cells and BASP1 expression. (B) The difference in infiltration levels of 24 kinds of immune cells between the high and low expression groups of BASP1. (C) Correlation between the relative abundance of 24 immune cells and the expression level of BASP1(Wilcoxon rank‐sum test; *n* = 375). (D) Relationship between six kinds of immune cell infiltration and somatic cell copy number of BASP1. (*P < 0.05; **P < 0.01; ***P < 0.001; ns, no significant difference).

Based on the results of the above enrichment analysis, we investigated the relationship between BASP1 expression and immune cell infiltration in depth. Immune cell infiltration analysis of the TIMER database showed that BASP1 expression in GC was favorably associated with infiltration of CD8+ and CD4+ T cells, macrophages, neutrophils, and dendritic cells (Fig. 5A). Subsequently, the relationship between BASP1 and infiltration of 24 immune cell subpopulations was further verified using the ssGSEA method, and the results demonstrated that the level of infiltration of all 20 immune cell subpopulations was higher in the BASP1 high expression group than in the BASP1 low expression group, except for Th17 cells, Th2 cells, NK CD56 bright cells, and T helper cells (Fig. 5B,C). In addition, somatic cell copy number analysis of the TIMER database suggested that the infiltration levels of B cells, CD8+ and CD4+ T cells, dendritic cells, neutrophils, macrophages, and monocytes associated with BASP1 were higher in the diploid/normal state than in the arm‐level deletion, arm‐level gain, and high amplification states (Fig. 5D). These results confirmed the important role of BASP1 in the immune microenvironment of GC.

**Fig. 6:**
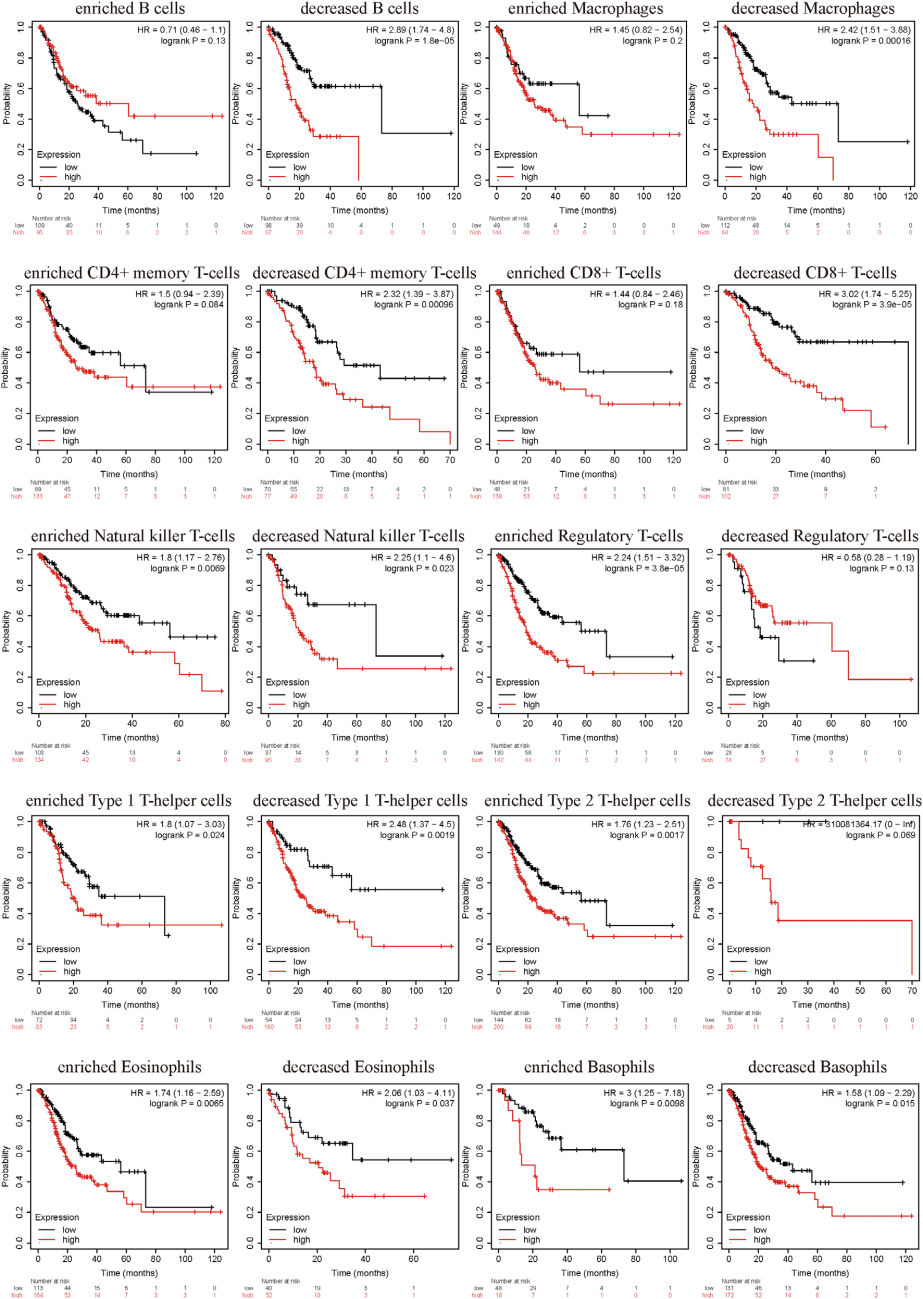
Kaplan–Meier survival curves of high and low expression of BASP1 in immune cell subgroups of GC patients with different degrees of infiltration.

Finally, we analyzed whether the degree of immune cell infiltration affected patient prognosis. The results revealed that patients with high BASP1 expression combined with reduced infiltration of B cells, macrophages, CD4+ memory T cells, CD8+ T cells, and increased infiltration of regulatory T cells and type 2T helper cells had a poorer overall prognosis (Fig. 6).

### Immune checkpoints and immune cell markers

**Fig. 7:**
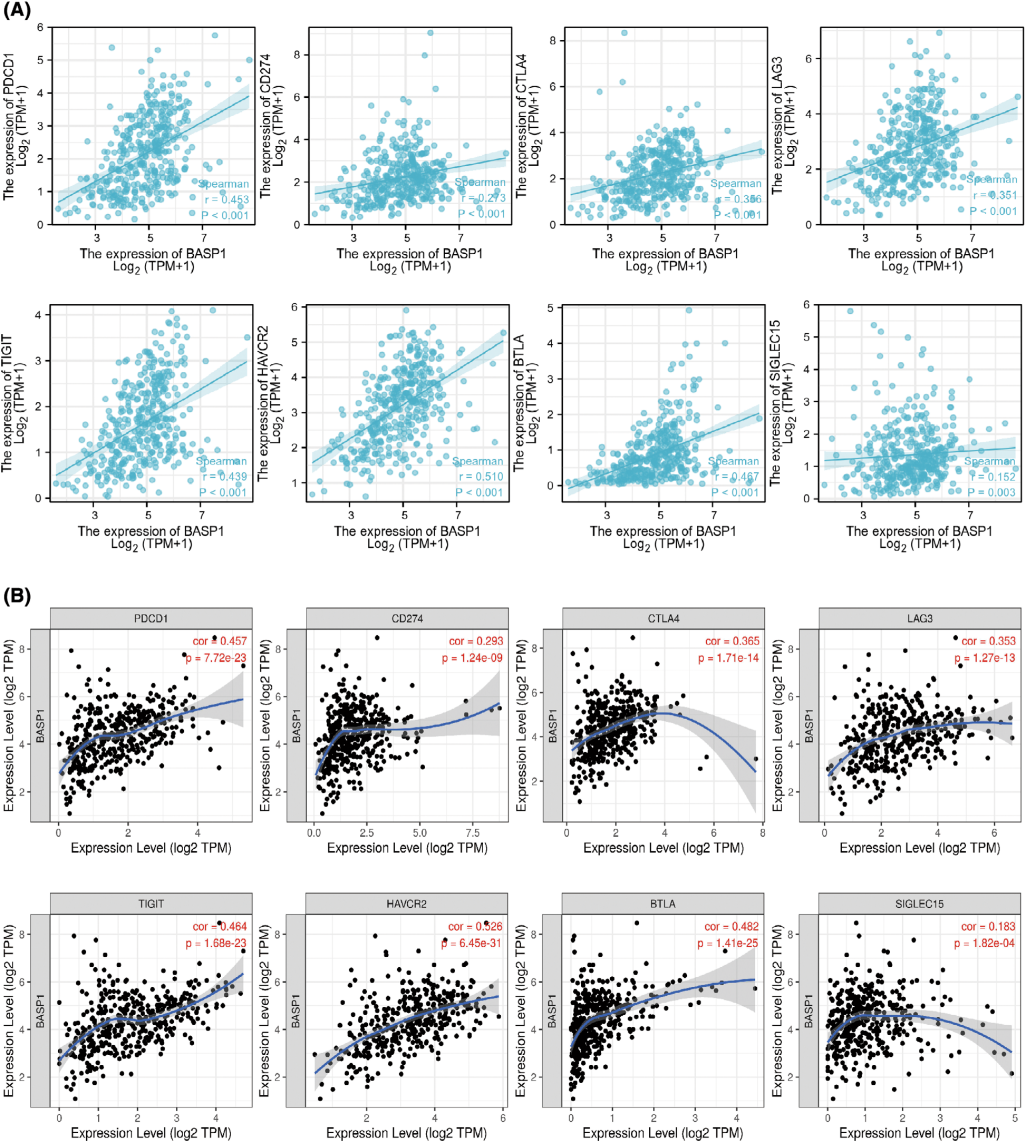
Correlation analysis of BASP1 and immune detection points. (A) Correlation between BASP1 expression and immune checkpoints in GC was analyzed based on TCGA database. (B) Correlation between BASP1 expression and immune detection points in GC was analyzed based on TIMER database.

To further explore the role of BASP1 in tumor immunity, we analyzed the correlation between BASP1 expression levels and those of immune checkpoints and immune cell surface markers in GC. An investigation based on TCGA database showed a significant positive correlation between BASP1 expression and the expression of PD‐1 (PDCD1), PD‐L1 (CD274), CTLA‐4, LAG‐3, TIGIT, Tim‐3 (HAVCR2), BTLA, and SIGLEC‐15 (Fig. 7A). Analysis of the TIMER database likewise confirmed this conclusion (Fig. 7B). It can be speculated that high expression of BASP1 may enhance the immune escape of GC cells. In addition, in the correlation analysis of immune cell markers, we found that BASP1 expression in GC was positively correlated with that of various surface markers of immune cells (Table 2), including CD19 and CD79A (B cells); CD4 (CD4+ T cells); CD8A and CD8B (CD8+ T cells); NOS2, IRF5, and PTGS2 (M1 macrophages); CD163, MS4A4A, and VSIG4 (M2 macrophages); CCR7 and ITGAM (neutrophils); HLA‐DPB1, HLA‐DRA, HLA‐DPA1, CD1C, NRP1, and ITGAX (dendritic cells); CD86 and CSF1R (monocytes); and KIR2DL1, KIR2DL3, KIR2DL4, KIR3DL1, KIR3DL2, and KIR3DL3 (natural killer T cells). Therefore, the use of adjuvant immunotherapy to specifically target BASP1 in GC may provide additional options for current treatments.

**Table 2:** Correlation between BASP1 expression and immune cell markers in GC.

Immune cell	Biomarker	*R* value	*P* value
B cell	CD19	0.36	1.1e−07
	CD79A	0.29	2.4e−05
CD4+ T cell	CD4	0.6	7.8e−22
CD8+ T cell	CD8A	0.33	1.1e−06
	CD8B	0.27	7.8e−05
M1 macrophage	NOS2	0.23	0.00076
	IRF5	0.42	1.8e−10
	PTGS2	0.35	2.3e−07
M2 macrophage	CD163	0.19	0.006
	MS4A4A	0.35	1.3e−07
	VSIG4	0.34	3.5e−07
Neutrophil	CEACAM8	0.06	0.39
	CCR7	0.59	5.4e−21
	ITGAM	0.41	3.8e−10
Dendritic cell	HLA‐DPB1	0.57	3.2e−19
	HLA‐DQB1	0.35	2.1e−07
	HLA‐DRA	0.55	4e−18
	HLA‐DPA1	0.58	2.2e−20
	CD1C	0.26	0.00013
	NRP1	0.45	6.1e−12
	ITGAX	0.54	1.5e−17
Monocyte	CD86	0.66	6.4e−78
	CSF1R	0.53	1.6e−46
Natural killer cell	KIR2DL1	0.35	7.1e−19
	KIR2DL3	0.41	4.3e−27
	KIR2DL4	0.36	8.5e−21
	KIR3DL1	0.36	3.4e‐20
	KIR3DL2	0.4	3.3e−25
	KIR3DL3	0.12	0.004

## Discussion

In recent years, despite the optimization of both diagnostic and therapeutic strategies for GC, patients still have an unfavorable prognosis, with a 5‐year survival rate of only 10% for advanced GC [36, 37]. Efforts are underway to elucidate the molecular mechanisms of GC carcinogenesis, leading to the development of therapeutic targets and prognostic biomarkers to alleviate this dilemma in patients with GC. BASP1 has been shown to act as an antioncogenic factor in a variety of cancers through its interaction with transcription factors such as WT1 and c‐myc. However, recent studies have found the opposite effect in some cancers. In cervical cancer, BASP1 promotes the proliferation and colony‐forming ability of tumor cells and accelerates the progression of the cell cycle [25]. In brain metastatic lung cancer, BASP1 reduces the degradation of EGFR protein via the ubiquitin–proteasome pathway and stabilizes EGFR protein, thereby promoting tumor progression [38]. The role of BASP1 in GC has rarely been reported, especially its potential role in the immune microenvironment of GC. In recent years, an increasing number of drugs have been developed and applied to the treatment of GC as research on immunotherapy and targets has intensified. Among them, the PD‐1 inhibitors nivolumab and pembrolizumab have been approved for monotherapy and combination therapy in advanced GC [39]. For patients with HER2‐positive GC, a combination of trastuzumab, nivolumab, and pembrolizumab is also entering the experimental phase [40]. Therefore, the development of new therapeutic targets is of great clinical and practical importance.

In the present study, we confirmed the high expression of BASP1 at the transcriptional level in GC using TCGA and GTEx data and obtained consistent conclusions by validation of the GEO database. Immunohistochemical results and western blotting experiments further confirmed this conclusion. Cox regression and Kaplan–Meier analyses showed that BASP1 could be used as an independent prognostic factor for patients with GC. The higher the pathological stage, histological grade, and T and N stages of GC, the higher the expression level of BASP1, which was closely correlated with poor prognosis. The nomogram constructed based on clinicopathological characteristics and indicators can predict the 1‐, 3‐, and 5‐year OS of patients with GC with high accuracy in clinical practice. In addition, GO, KEGG, and GESA enrichment analyses suggested that BASP1 may be involved in ‘regulation of T‐cell activation’, ‘primary immunodeficiency’, and other processes that are highly related to the immune response. In the analysis of immune cell infiltration, we also found that BASP1 expression was positively correlated with the infiltration of most immune cells. Previous research has demonstrated that the expression of BASP1 is higher in germinal center B cells generated by T‐cell‐dependent (TD) immune responses than in germinal center B cells generated by T‐independent (TI) immune responses [41]. However, germinal center B formed in the TI reaction has a lifespan of only a few days [42]. This indicates that high BASP1 expression is conducive to the survival of immune cells and plays an important role in immune function. Interestingly, the massive immune cell infiltration caused by high BASP1 expression did not seem to benefit the survival of patients with GC, and in the survival analysis, the higher the BASP1 expression, the shorter the OS of the patients. In the study of immune checkpoint correlation, we discovered that BASP1 expression was positively linked with a number of immunological checkpoints, such as PD‐1, PD‐L1, and CTLA‐4, which may increase the occurrence of immune escape from tumor cells. Therefore, the role and specific mechanisms of BASP1 in the immune microenvironment of GC need to be further explored in future studies.

## Conclusion

In conclusion, this study suggests that BASP1 is highly expressed in GC and can be used as an independent indicator to predict poor OS in patients with GC. BASP1 is highly correlated with immune processes, and its expression is positively correlated with the degree of immune cell infiltration, immune checkpoints, and immune cell markers. However, this study is based on bioinformatics and clinical samples and has certain limitations. The mechanism by which BASP1 regulates tumor immunity needs to be further verified *in vivo* and *in vitro*.

## Conflict of interest

The authors declare no conflict of interest.

### Peer review

The peer review history for this article is available at https://www.webofscience.com/api/gateway/wos/peer‐review/10.1002/2211‐5463.13654.

## Author contributions

MZ and TongW contributed to the study's conception and design. Material preparation, data collection, and analysis were performed by XL and LZ. The first draft of the manuscript was written by TaoW.

## Data Availability

The original contributions presented in the study are included in the article; further inquiries can be directed to the corresponding author.
